# Dual Autoencoder Network with Separable Convolutional Layers for Denoising and Deblurring Images

**DOI:** 10.3390/jimaging8090250

**Published:** 2022-09-13

**Authors:** Elena Solovyeva, Ali Abdullah

**Affiliations:** Department of Electrical Engineering Theory, Saint Petersburg Electrotechnical University “LETI”, 197022 St. Petersburg, Russia

**Keywords:** machine learning, image processing, computer vision, image denoising, autoencoder, dual autoencoder, convolutional neural network, separable convolutional neural network, deep learning, non-linear model

## Abstract

A dual autoencoder employing separable convolutional layers for image denoising and deblurring is represented. Combining two autoencoders is presented to gain higher accuracy and simultaneously reduce the complexity of neural network parameters by using separable convolutional layers. In the proposed structure of the dual autoencoder, the first autoencoder aims to denoise the image, while the second one aims to enhance the quality of the denoised image. The research includes Gaussian noise (Gaussian blur), Poisson noise, speckle noise, and random impulse noise. The advantages of the proposed neural network are the number reduction in the trainable parameters and the increase in the similarity between the denoised or deblurred image and the original one. The similarity is increased by decreasing the main square error and increasing the structural similarity index. The advantages of a dual autoencoder network with separable convolutional layers are demonstrated by a comparison of the proposed network with a convolutional autoencoder and dual convolutional autoencoder.

## 1. Introduction

After the invention of the camera in 1816, images became a significant information-saving and data-saving unit in our lives. The use of images influenced all the fields of human knowledge, from history and art to medicine and technology and many others. However, one of the main disadvantages of images in physical and digital forms is their lack of resistance to noise and impact. In addition, with the development of digital cameras, new types of noises that can affect an image appeared; therefore, noise reduction in image processing and computer vision started to get more attention from researchers due to its importance, and image denoising became a significant area of research. Many models are built by means of algorithms that identify connections among picture pixels and generate predictions about the value of an individual pixel based on the attributes of the image [1]. Image denoising is applied in the following:

- In image restoration, which is the methodology of reaching the original image from a distorted noisy image. Image restoration is accomplished by inverting the process that blurs or destroys the parts of the original image [2,3,4,5,6,7].
- In visual tracking, which is the process of tracking objects, animals, or humans using cameras, the tracking environment could have a lot of noise sources caused by natural reasons such as weather like heat or by stimulated reasons such as radiation in nuclear stations [8,9].
- In image registration, which is the technique of overlaying multiple images to be examined, data from several images have to be combined to gather information using different sensors and those could be exploited to noise [10,11].
- In image segmentation, in which noise in pixels can appear when dividing a single digital image into multiple portions called image segments or image regions to facilitate or modify the image presentation into a form, which is more significant and more comfortable to investigate and analyze [12,13].

Significantly, artificial intelligence and machine learning found origins in image denoising because images are one of the main sources of information that can arise from different origins, including the internet, medical archives, historian archives, social media, reviews, etc. Since images are not resilient to noise, understanding information from distorted or noised images would be challenging and difficult, yet after the development of deep learning, image denoising is developing and allowing us to be able to restore the original images and to take a piece of meaningful information from it with minimal effort. Many algorithms and approaches are used to achieve image denoising, such as:

1. Spatial domain filtering is a method for extracting noise by calculating the gray value for the current pixel, relying on itself and the surrounding pixels in the original picture. In general, linear and non-linear filters are the two primary classes of filters [5,6,7,14,15,16]. Linear filters in the spatial domain removed noise, but they failed to maintain image consistencies. For example, the Wiener filter was used for Gaussian noise (Gaussian blur or Gaussian smoothing) reduction to overcome the disadvantage of the mean filter because it can overfit the smoothness of an image with high noise [17,18]. However, the Wiener filter sometimes caused the sharp edges in images to be blurred. Non-linear filters, such as the median filter, weighted median filter, and bilateral filter, can reduce noise without any unique identification due to their edge-preserving characteristic [19,20,21]. This characteristic indicates that the intensity value of each pixel is substituted with a weighted average of the intensity values from nearby pixels rather than a single intensity value.

2. Total variation is a method founded on the principle that the integral of the absolute image gradient increases when the images include excessive details; therefore, it has a high total variation. As a result, reducing a noisy image’s overall variance to a near value to the original image removes extraneous detail while maintaining critical elements such as edges. The key benefit of this approach over the previous methods is that it decreases noise while at the same time maintaining the borders of the picture [22,23,24].

3. Non-local means, in this technique, the main difference from the previous methods is that it depends, unlike them, on all the pixels in the image in calculating the mean. After that, it weights the mean of all the pixels according to their similarity with the target pixel, resulting in significantly better post-filtering clarity and less image information loss [25,26,27].

4. Data-adaptive transform, which is a technique to improve artifact removal based on the transform domain approach using a blend of data-adaptive and non-data-adaptive transform domains [28]. The removal of artifacts from the scalp electrocardiogram is critical for both automatic and visual examinations of underlying brainwave activity [29].

5. Non-data-adaptive transform is a technique that may be separated into two domains: the spatial-frequency domain and wavelet domain, which is a methodology for transform domain filtering approaches [30]. Low-pass filtering is a spatial-frequency domain filtering technique that works by producing a frequency-domain filter that allows all frequencies below a cut-off frequency to pass through while decreasing all frequencies beyond the cut-off frequency. In contrast, a scale-space representation is created by the use of the wavelet transform [31].

6. A significant area of research is image denoising where many models are built on algorithms that identify connections among picture pixels and generate predictions about the value of an individual pixel based on the attributes of the image. This challenge may be thought of as a supervised learning problem in which each noisy image represents an input, and the intended result is the original shape of the image [32]. This supervised learning model analyzes the observations or input images and provides a solid representation of the connection between the input and output. Research in image processing and computer vision with diverse experiences has started using deep neural networks to solve a vast range of tasks in these fields, including image classifications, image recognition, face detection, face recognition, emotion recognition, and now image denoising [33,34,35,36,37,38]. This breakthrough made it possible to use deep neural networks to examine the picture denoising problem. A convolutional neural network (CNN) has the ability to learn noise patterns due to its property of feature extraction; therefore, by constructing a model of multiple layers of CNN, this model will be able to learn these patterns and modify its weights through the training process on big datasets to cancel these noise patterns. Therefore, many researchers in image denoising used convolution neural networks as a base for their models. The main modification for convolution layers is to connect them with other layers such as batch normalization, Relu layers, and Tanh layers to build enhancement blocks, sparse blocks, and restoration blocks in the models, which achieve the denoising process but with a high computational cost. In [39], a feedforward architecture of four stages of filtering to denoise mixed Gaussian-impulse noise was represented. The first stage was a rank order filter formed from existing filters and the adaptive switching weight mean filter, then three stages of the CNN layers for the rest of the filtering process. In [40], two modifications to the use of convolution neural networks were proposed. The first modification combined convolution neural networks, batch normalization, and Relu layers to build a denoise model of four blocks: sparse block, feature enhancement block, attention block, and reconstruction block. The second modification was the feedforward model of five layers of convolution neural networks with Relu layers to get a denoised image and then enhance it using a pretrained semantic segmentation model (SegNet). In [41], a pseudo convolutional neural network model was used to preprocess the image before denoising. The pseudo convolutional neural network model consisted of three layers (a convolution layer, a pooling layer, and a filter layer) to get a preprocessed image. After getting the preprocessed image, the model that combined convolution layers, Relu layers, and batch normalization was used to get the noise from the noisy image and then subtract it from the preprocessed image to get the denoised image. 

7. Many researchers have lately started to use autoencoders built with convolutional neural networks (CNNs) to reduce the noise in images [42,43,44,45,46]. Deep neural networks are increasingly being used to handle a wide range of problems in image processing and computer vision research, including picture classifications, image identification, face detection, face recognition, emotion recognition, and even image denoising. This breakthrough made it possible to examine the topic of picture denoising using deep neural networks. To minimize picture noise, several researchers have recently begun to employ autoencoders constructed using convolutional neural networks. Autoencoders with convolution layers were developed from the previous use of autoencoders with fully connected layers. Since this development, the main modifications for using the autoencoder are to connect its output with other outputs from blocks of convolution neural networks or other types of neural networks, then sum these different outputs to get the final output to enhance the output of the autoencoder. In [42], two models were presented to achieve the denoise process. The first model was a combination of convolution neural layers with max-pooling followed by multiple layers of fully connected layers. The second model was an autoencoder with convolution neural networks and pooling layers, where their results showed the benefits of using an autoencoder in image denoising over convolution neural networks and fully connected layers without autoencoders. In [43], three separated structures were suggested that combine autoencoders with feedforward convolution neural networks, pooling layers, and fully connected layers. Each structure was trained independently, and the final output was a sum of the three outputs of each structure. In [44], two structures of deep neural networks were proposed; the first structure was convolution layers to extract global and local noise. In comparison, the second structure was an autoencoder with pixel up-sampling convolution layers to remove the noise features from the image. This dual structure was used to denoise hyperspectral images and work on wavelet transformed bands. In [45], the system consisted of four blocks: a preprocessing block, preparing synthetic image block, stacked convolutional autoencoder block, and validation measure block. Therefore, after the image was preprocessed and prepared, it entered the stacked convolutional autoencoder (SCAE) block, where the learning process took place to remove the noise, and then it was validated in the validation measure block. In [46], the denoising system consisted of two stages. The first stage was the fast region-based convolutional neural networks (fast R-CNNs) to restrict the areas in the images that contained the noise. The second stage was denoising the images using a model of shared layers of convolution neural networks followed by two structures of deep denoised autoencoder (DDAE) and deep material classifier (DMC). The DMC consisted of two convolution layers followed by fully connected layers and shared the first two convolution layers with the autoencoder DDAE, where the DDAE consisted of fully connected layers followed by three deconvolution layers. 

In general, these modifications gave the use of the autoencoder the ability to extract features from non-linear processes and perform non-linear transformations image denoising. However, the main disadvantage was an increase in the computational cost and the time of training and performing. In this study, the first modification to the use of an autoencoder is to build a dual autoencoder. The second modification is to use separable convolution neural networks instead of convolution neural networks. These modifications give our model the additional advantages of increasing the accuracy of the image restoration, reducing the training parameters, decreasing the training and performing time of the autoencoder’s network, and saving memory. 

Thus, we can emphasize the following pros for the proposed methodology. First, we use a special type of neural network, namely, an autoencoder network, which extracts features from non-linear processes and transformations by means of dimensionality reduction when compressing the data and information retrievals when decompressing the data. Second, the dual autoencoder network ensures a higher accuracy of denoising images in comparison with one autoencoder network. Third, the use of separable convolution neural networks in dual autoencoders results in decreasing the number of networks parameters and, consequently, the amounts of the calculation time and occupied memory. The con for methodology, which is characteristic of autoencoders, consists in the difficulty to reach the high image restoration quality under a large number of small features.

The paper contains six sections. The significance of image denoising and its application fields, the main algorithms, and approaches are used to achieve the image denoising mentioned in the introduction. The main noise that can affect an image is described in Section 2. The mathematical model of autoencoder and dual autoencoder using convolutional neural networks and separable convolutional neural networks (SCNNs) is represented in Section 3. The results of image denoising, their comparison, and corresponding conclusions are described in Section 4, Section 5 and Section 6.

## 2. Basic Types of Noise on Images

Noise is generally described as an accidental variation in brightness or pixel information, and it is often created by accident or by the limitations of the image sensor or camera. Improper environmental circumstances can also create noise. These problems are repeatedly unavoidable in real systems, which increases the importance of image denoising in image processing and computer vision. Gaussian noise (Gaussian blur), Poisson noise, Speckle noise, and impulse noise are considered the main noises that can affect an image.

### 2.1. Gaussian Noise

The term “Gaussian noise,” anointed after Carl Friedrich Gauss, refers to a sort of signal noise with a probability density function equivalent to that of the normal distribution (Gaussian distribution) [47], which means the potential values of the noise are dispersed according to the Gaussian distribution depending on Equation (1). The normal distribution formula is given as the following:

(1) $$G(x)=\frac{1}{\sigma \sqrt{2\pi }}e^{-\frac{{\left(x-\mu \right)}^{2}}{2\sigma^{2}}},$$

where G(x) is the normal distribution function; x is the input pixel value; σ is the standard deviation; and μ is the mean of the values of the input pixels surrounding a current pixel. Sensor noise caused by low light or extreme temperature, as well as transmission noise such as electrical circuit noise, are the main origins of Gaussian noise in digital photographs. Gaussian noise may be minimized using a spatial filter in digital image processing; however, when smoothing a picture, an unwanted byproduct may be the blurring of fine-scaled image edges and features, which correspond to blocked high frequencies [48]. Mean filtering, median filtering, and Gaussian smoothing are three common spatial filtering techniques for noise reduction [49].

### 2.2. Poisson Noise

The quantized character of light and the independence of photon detections make Poisson noise a primary kind of uncertainty related to light measurement. Except in low-light settings, its predicted magnitude is signal-dependent, and it is the most common cause of picture noise [50]. The discrete character of electric charge causes shot noise in electronics. Shot noise may also be seen in photon counting in optical systems, where it is linked to light’s particle nature [51]. Each photon observation can be considered a separate event with a randomized distribution pattern. The discrete probability distribution can define the Poisson process:

(2) $$P(x)=\frac{e^{-\lambda t}{\left(\lambda t\right)}^{x}}{x!},$$

where P(x) is the discrete probability distribution; x is the total number of photons detected by a sensor element during a period of time t; λ is the amount of photons anticipated per unit time period; and t is the time interval. 

Equation (2) is a typical Poisson distribution with the anticipated photoelectric effect number as the rate parameter λt. Poisson noise is the uncertainty indicated by this distribution. Typically, this estimate is highly accurate. Poisson noise is usually controlled by other signal-independent forms of noise for lower photon counts. Photon noise is a low constraint on the uncertainty of measuring light since it is generated from the nature of the signal. Any test would be prone to Poisson noise even under perfect imaging circumstances, devoid of all other sensor-based causes of noise [52]. Imaging is considered photon-limited when Poisson noise is the only substantial source of uncertainty, which is frequent in bright photon-rich situations.

### 2.3. Speckle Noise

Speckle is a particulate disturbance that occurs naturally in radars, synthetic aperture radar, medical ultrasonography, and optical coherent tomography pictures and decreases their clarity. On the frequency scale, most artificial and natural surfaces are highly harsh. The common interference phenomena of speckle affect images acquired from these surfaces by coherent imaging methods such as laser, synthetic aperture radar, and ultrasound. If we describe our reflectivity function as an array of scatterers, we can understand where this behavior comes from. Because of the finite resolution, it is transmitted at any given moment from a large number of potential distributions inside the resolution cell. These dispersed signals add coherency. Depending on the relative phases of each distributed signal, they add positively or harmfully. These constructive and destructive interference patterns, depicted as bright and dark spots in the picture, cause speckles. Whenever the diffuser’s radiation pattern is considered the primary important parameter for producing an image during the imaging process, and the change in Radom light phases during the reflection process is not taken into account, speckles in pictures can be treated completely as noise [53,54]. The phase function of diffused lights may be thought of as a non-correlation random field with an equal distribution between 0 and π where the optically surface may be thought of as a collection of several random diffused patches described in Equation (3). Evaluating the features of a true speckle pattern reveals that it invariably comprises signal-dependent disturbances such as speckle noise. The speckle pattern mathematical model can be described as the following [55]:

(3) $$g(x,y)=f(x,y)+r[f(x,y)]\times [n_{1}(x,y)+n_{2}(x,y)]+n_{3}(x,y)+p(x,y),$$

where g(x,y) is the speckle pattern; x,y is the dimension of the image; f(x,n) is the intensity of the original image; $n_{2}(x,y)$ and $n_{3}(x,y)$ are statistically independent and signal-independent from one another, random Gauss distribution noise with zero mean; $n_{1}(x,y)$ is a random noise that has some spatial correlation, the degree of which is directly related to the amplitude of the imaging system’s incoherent point spread function; p(x,y) is the pulse noise whose amplitude probabilistic density is evenly distributed over the image’s dynamic range; and r[f(x,y)] is the original image pattern’s compound function.

### 2.4. Impulse Noise

Salt and pepper noise is another name for impulse noise where sharp and rapid disruptions in the visual signal might create this noise. It manifests as a scattering of white and black pixels [5,6,7,56,57,58]. Transmitting data faults, memory cell failures, and analog-to-digital converter flaws can all produce this issue. It takes the shape of white and black pixels that appear at random and can drastically degrade image quality. Noisy pixels in photos degraded by salt and pepper noise are alternately adjusted to the minimum and highest intensity values, giving the image a “salt and pepper” look described in Equation (4). Unaffected pixels, however, are never affected [59,60]. The following mathematical equation may be used to express the impulse noise:

(4) $$F(x,y)=\left\{\begin{array}{l} s_{\min}\;\mathrm{with}\;\mathrm{probability}\;p\\ s_{\max\;}\;\mathrm{with}\;\mathrm{probability}\;q\\ I(x,y)\;\mathrm{with}\;\mathrm{probability}\;1-p-q \end{array}\right.,$$

where F(x,y) is the noisy image matrix with impulse noise with dimensions (x,y); $s_{\min}$ is the minimum value of the pixels in the original image I(x,y); $s_{\max\;}$ is the maximum value of the pixels in the original image I(x,y); and I(x,y) is the original image matrix without the impulse noise.

## 3. Dual Autoencoder with Convolutional and Separable Convolutional Neural Networks

Autoencoders are a kind of artificial neural network design utilized to develop efficient coding for unlabeled input. In the unsupervised learning process, the architecture of neural networks is such that we may place a bottleneck in the network, which pushes the network to represent the original input in a compressed manner [61]. Assuming that each of the input attributes is completely independent of the others, this compression and reconstruction operation will be quite challenging. In the case of some structure in the data such as the correlations between input characteristics, this structure may be learned and used to the advantage of the user when driving the input through the bottleneck in the network. Autoencoders are primarily dimensionality reduction (or compression) algorithms with several critical characteristics, including the following: 

1- They are data-specific. Autoencoders can only significantly compress data similar to the data on which they were previously trained. Because they learn characteristics particular to the training data, they differ from a basic data compression technique such as zip in that they learn features specific to the training data [62]. This means that a landscape photo cannot be compressed by an autoencoder that has been trained on handwritten numbers. 

2- They are loosely encoding. The output of the autoencoder will not be an exact replica of the input; rather, it will be a close but degraded copy of the original [63]. 

3- They are an unsupervised learning technique. Autoencoders are classified as an unsupervised learning technique because they do not require explicit labels to be trained on to be effective [60]. To be more specific, they are self-supervised in the sense that they use the training data they are given to make their own labels.

In order to construct an autoencoder, three components are required: an encoding method, a decoding method, and a loss function for comparing the output to the target signal.

**Figure 1:**
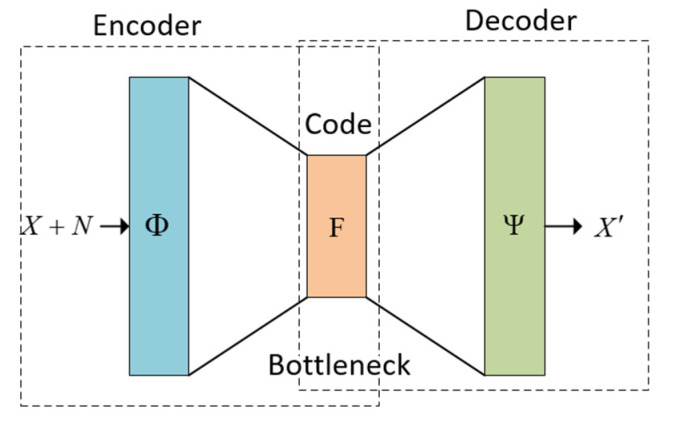
The autoencoder structure.

The autoencoder model achieves a balance between the properties and characteristics listed previously. To properly rebuild the scene, it must pay close attention to the input signals. A bottleneck (in Figure 1) restricts the amount of information that can be transferred throughout the whole network due to congestion. By punishing the network depending on the reconstruction error, our model may learn how to restore the original input from an “encoded” state in the most efficient manner feasible [64]. The autoencoder structure is shown in Figure 1 and the model is described as Equation (5):

(5) $$\begin{array}{ll} \Phi :X+N\to \;F\\ \Psi :F\to X^{\prime } & ,\\ Loss=\underset{\Phi ,\Psi }{\arg\min}||X-Y|| \end{array}$$

where Φ is the encoder function that maps the noisy image into the space F that represents the bottleneck; X is the matrix of the original image; N is the noise matrix added to the original image; F describes the space of matrices in the bottleneck; Ψ is the decoder function that maps the space F of the bottleneck into the output; $X^{\prime }$ is the output matrix of the autoencoder; and Loss represents the loss function that aims at minimizing the error between the desired output X and the current output $X^{\prime }$.

**Figure 2:**
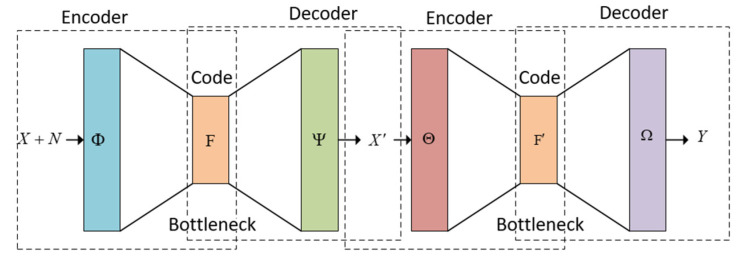
The dual autoencoder structure.

Based on the autoencoder structure, a dual autoencoder is proposed to denoise images. The dual autoencoder structure is shown in Figure 2.

As can be seen from Figure 2, there are four kinds of mapping: two for denoising the image and two for enhancing the quality of the denoised image. The model of the dual autoencoder is described as the following:

(6) $$\begin{array}{ll} \Phi :X+N\to \;F\\ \Psi :F\to X^{\prime }\\ \Theta :X^{\prime }\to F^{\prime } & ,\\ \Omega :F^{\prime }\to Y\\ Loss=\underset{\Phi ,\Psi ,\Theta ,\Omega }{\arg\min}||X-Y|| \end{array}$$

where X is the matrix of the original image; N is the noise matrix added to the original image; $X^{\prime }$ is the output matrix of the first autoencoder; Y is the output matrix of the second autoencoder; Φ and Θ are the encoder functions of the first and second autoencoders, which map the matrix (X+N) into the space F of matrices and the matrix $X^{\prime }$ into the space $F^{\prime }$, correspondingly; Ψ and Ω are the decoder functions of the first and second autoencoders, which map the space F into the matrix $X^{\prime }$ and the space $F^{\prime }$ into the output Y, accordingly; and Loss represents the loss function that aims at minimizing the error between the desired output X and the current output Y.

In the case of deep autoencoders, we must additionally consider the capabilities of our encoder and decoder models, respectively. Ensuring that the autoencoder model is not just learning an effective technique to remember the training data is critical. Otherwise, the model will fail. Similarly, to supervised learning concerns, we may use various regularization techniques in the network to promote excellent generalization properties. Because of the input data, a sparse autoencoder is constrained to activate sections of the network selectively. In contrast, a complete autoencoder is forced to employ the whole network for every observation. This implies that we may pick a latent state representation (i.e., encoding dimensionality) that makes sense in the data context while still enforcing regularization via the sparsity restriction on the latent state representation. One consequence is that we can allow our network to sensitize certain hidden layer nodes to specific properties of the raw data as a result [65]. 

**Figure 3:**
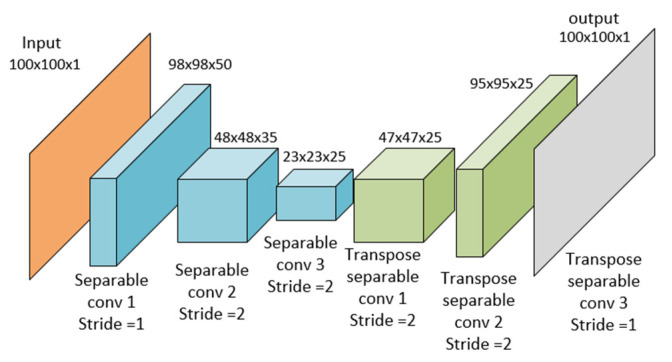
The neural architecture of the autoencoder.

**Figure 4:**
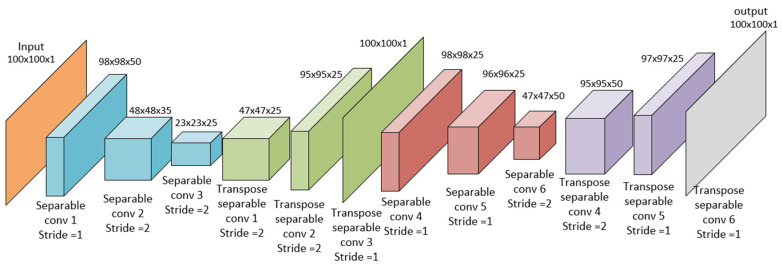
The neural architecture of the dual autoencoder.

Autoencoders can be built from different kinds of neural networks, but since the aim of this research is image denoise, they were built using separable convolutional neural networks and later with a convolutional neural network (see Figure 3 and Figure 4). The neural architecture of the autoencoder depicted in Figure 3 corresponds to the structure in Figure 1, and the neural architecture of the dual autoencoder in Figure 4 corresponds to the structure in Figure 2.

As shown in Figure 3 and Figure 4, the number of kernels in the last layer gives the ability of this model of autoencoder to denoise colored images or gray images. In our experiment, we used a transpose convolution layer with 100 × 100 × 1; therefore, the denoise process is on gray scale images. However, when changing the parameters in the last layer to three kernels instead of one, the same model will be able to denoise colored images. The noisy image of size 100 × 100 × 1 is the input of the autoencoder illustrated in orange. Then, the encoding presented in blue takes place by reducing the input size 100 × 100 to 23 × 23. The encoder marked in blue comprises three layers, and every of them is a separable convolutional neural network. These networks have filters 3 × 3 × 50, 3 × 3 × 35, and 3 × 3 × 25 with 50, 35, and 25 kernels, correspondingly. Then, the decoding process is shown in green; this process raises the output of each layer to reach the 100 × 100 × 1 image similar to the input one but with noise reduction. Like Figure 2, the autoencoder in Figure 3 is added to another autoencoder with the same architecture but with the softer size decreased to enhance and denoise. The encoding process is described in Equations (7)–(10), whereas the decoding process is described in Equations (11)–(14) for using both convolutional and separable convolutional neural networks. The purpose of building the autoencoder and dual autoencoder using separable convolutional neural networks and convolutional neural networks is to compare between the performance of the two types of neural network knowing that separable convolutional neural networks achieve a convolution product with a smaller number of learnable parameters. The capability of the convolution layer to extract data with local characteristics is directly connected to the complexity of this layer. Because of its consistent structure, it is more analogous to the neural networks seen in the human brain. It also makes the model easier to understand because it reduces the total number of weights. Padding is added to the input matrix right before the product is finished so that it can accommodate all of the inputs. Padding parameters can have two values, zero or one. The primary use of the padding parameter is to resolve how many elements to add to the original image or matrix to increase its size in a way that makes all its pixels reachable from all edges. This allows the output to have a smaller size or a bigger size with the deconvolution product. The “stride” is the step taken by the kernel or the filter to make the product. The term “stride” refers to the stage in the convolution product process. When performing a convolution layer on two matrices, the kernel or filter is used to operate and update the learnable parameters for the layer. Separable convolution layers, however, are fully associated with dimensions since the kernel matrix is split into a product between a row and a column that produces this matrix, which reduces the trainable parameters in such a way that a kernel matrix K with the size of (f×f) can be represented with two partial kernels $K_{1}$ and $K_{2}$ with the size of (f×1) and (1×f). In this way, for example, the learnable parameters of a kernel with size 3×3 will be reduced to six parameters instead of nine [66]. The main equation that describes the convolution product of the ordinary convolution layer is described in the following equation:

(7) $$\mathrm{Conv}(X,K)=\varphi (\sum_{i=1}^{n_{h}}\sum_{j=1}^{n_{w}}X_{i,j}K+b),$$

(8) $$\mathrm{Dim}\left(\mathrm{Conv}(X,K)\right)=\left(\frac{n_{h}+2p-f}{s}+1,\frac{n_{w}+2p-f}{s}+1\right),$$

where Conv(X,K) represents the convolution product of matrix X of size $\left(n_{h}\times n_{w}\right)$ with a kernal matrix K of size (f×f); φ is the sigmoid activation function; *i* and *j* are counters; $X_{i,j}$ is a sub-matrix of size (f×f) of the input matrix with its padding; b is the bias; $\mathrm{Dim}\left(\mathrm{Conv}(X,K)\right)$ is the dimension of the results of the convolution product; $n_{h}$ and $n_{w}$ are the elements of the matrix X dimension; p is the padding parameter; s is the number of strides; and f is the kernel size.

When using the separable convolution, Equation (7) is represented in two convolution products: one is with the partial kernel $K_{1}$ of size (f×1) and the second is with the partial kernel $K_{2}$ of size (1×f). In this way, the computational cost represented by the learning parameters and the time of learning will be reduced while getting the same and sometimes enhanced results. The equation describing the separable convolution product is described as the following:

(9) $$\bar{X}=\mathrm{Conv}(X,K_{1})=\sum_{i=1}^{n_{h}}\sum_{j=1}^{n_{w}}X_{i,j}K_{1},$$

(10) $$\mathrm{Conv}(\bar{X},K_{2})=\sum_{i=1}^{(n_{h}+2p-f)/s+1}\sum_{j=1}^{n_{w}}K_{2}{\bar{X}}_{i,j},$$

where $\bar{X}$ is the output of the convolution product $\mathrm{Conv}(X,K_{1})$ of the input matrix X with the kernel $K_{1}$ of size (f×1), giving a matrix of size $\left(\left((n_{h}+2p-f)/s+1\right)\times n_{w}\right)$; p is the padding parameter; s is the number of strides; $X_{i,j}$ is a column from the input matrix in the position (i,j); $\mathrm{Conv}(\bar{X},K_{2})$ is the convolution product between matrix $\bar{X}$ and the kernel $K_{2}$ of size (1×f); and ${\bar{X}}_{i,j}$ is a row from the matrix $\bar{X}$ in the position (i,j).

When we use a separable convolutional neural network, Equation number (7) is represented by Equations (9) and (10). In contrast, Equation (8), which illustrates the size of the output of the convolution product, will stay the same. The encoding process is described with the Equations (7), (9) and (10), whereas the decoding process is understood to be the transpose of the convolution product, which means we took the transpose of Equations (7), (9) and (10) to achieve the decoding process of the autoencoder. Mathematically, instead of multiplying a partial part from the input matrix X of size $\left(n_{h}\times n_{w}\right)$ by the K of size (f×f) in Equation (7) or by two sub-kernels $K_{1}$ of size (f×1) and $K_{2}$ of size (1×f) in Equation (9) and (10), we multiply each value in the input matrix of size $\left(n_{h}\times n_{w}\right)$ by the kernel of size (f×f) to produce a (f×f) matrix or by $K_{1}$ and $K_{2}$ to produce a column of size (f×1) or a row of size (1×f). Then, we combine all the resulting matrices or vectors according to the initial positions in the input layer and add the overlaid values together with an output padding parameter $p_{o}$ [67]. Therefore, we can describe the transpose of Equation (7) as the following:

(11) $$\mathrm{TransConv}(X,K)=\varphi (\sum_{i=1}^{n_{h}}\sum_{j=1}^{n_{w}}x_{i,j}K+b),$$

(12) $$\mathrm{Dim}\left(\mathrm{TransConv}(X,K)\right)=\left((n_{h}-1)\times s+f-2p+p_{o},(n_{w}-1)\times s+f-2p+p_{o}\right),$$

where TransConv(X,K) represents the transpose convolution product of matrix X of size $\left(n_{h}\times n_{w}\right)$ with a kernel matrix K of size (f×f); φ is the activation function; *i* and *j* are counters; $x_{i,j}$ is the pixel in matrix X, after we add padding and bias b; $\mathrm{Dim}\left(\mathrm{TransConv}(X,K)\right)$ is the dimension of the results of the transpose convolution product; $n_{h}$ and $n_{w}$ are the elements of the dimension of the matrix X; p is the padding parameter; s is the number of strides; $p_{o}$ is the output padding parameter; and f is the kernel size.

In the same way, the transpose of the separable convolution product in Equations (9) and (10) can be represented as the following:

(13) $$\bar{X}=\mathrm{TransConv}(X,K_{1})=\sum_{i=1}^{n_{h}}\sum_{j=1}^{n_{w}}x_{i,j}K_{1},$$

(14) $$\mathrm{TransConv}(\bar{X},K_{2})=\sum_{i=1}^{(n_{h}-1)\times s+f-2p+p_{o}}\sum_{j=1}^{n_{w}}K_{2}{\bar{x}}_{i,j},$$

where $\bar{X}$ is the output of the transpose convolution product $\mathrm{TransConv}(X,K_{1})$ of the input pixel in the position (*i*, *j*) in the matrix X with the kernel $K_{1}$ of size (f×1), the output is a matrix of size $\left(((n_{h}-1)\times s+f-2p+p_{o})\times n_{w}\right)$; p is the padding parameter; $p_{o}$ is the output padding parameter; s is the number of strides; $x_{i,j}$ is the pixel from the input matrix in the position (i,j); $\mathrm{Conv}(\bar{X},K_{2})$ is the convolution product between matrix $\bar{X}$ and the kernel $K_{2}$ of size (1×f); and ${\bar{x}}_{i,j}$ is the pixel of matrix $\bar{X}$ in the position (i,j).

From Equations (7)–(10), we can understand the encoding process where the size of the input image has been reduced; from Equations (11)–(14), we can understand the decoding process, where the size of the resulting output is increased till it matches the original size of the input image. This process of reducing the size in the encoding allows us to remove pixels from the input noisy image and later restore those pixels in the decoding process in a way that cancels the noise and predicts the original image pixels before adding the different noises. 

## 4. Results of Image Denoising

In our practical experiment, we used Python 3.9.7 and Tensorflow 2.6.0 with a Pycharm.3.1 2019 environment on an Asus ZenBook Core i7 2.8 GHz 32 G RAM.

The used dataset was 99,534 images of size 100 × 100 from a combination of famous datasets of people’s faces: IMDB-WIKI, FER-2013, KDEF, and AffectNet [68,69,70,71]. We chose these datasets because face images easily get affected by different types of noises, and many technologies have started to rely on face recognition and face detection, adding more importance to this type of image in the present and future [72]. Additionally, this combination of the dataset has various races, ages, and genders. In the training process, the combined dataset was divided into 80,000 images for training and the rest for testing. We started by adding the four kinds of noise using the scikit-image library in Python. The amount of Gaussian noise (Gaussian blur) was between medium and high with a standard deviation of 40, Poisson noise was generated from each image value using the scikit-image library, the speckles’ noise amount was 20%, and the amount of salt and pepper noise was 30%. After adding these noises to the combined dataset, we got four datasets, one for each of the previous noise types. These datasets were used as an input to the autoencoder and dual autoencoder, and the outputs of the autoencoder and dual autoencoder were compared with the original dataset without the noise using the structural similarity index, which is known as an estimation to compare the similarity of two images and is described by the following Equation [73]:

(15) $$\mathrm{SSIM}(X,Y)=\frac{\left(2\mu_{X}\mu_{Y}+{\left(0.01l\right)}^{2})+(2\sigma_{XY}+{\left(0.03l\right)}^{2}\right)}{\left(\mu_{X}{}^{2}+\mu_{Y}{}^{2}+{\left(0.01l\right)}^{2})(\sigma_{X}{}^{2}+\sigma_{Y}{}^{2}+{\left(0.03l\right)}^{2}\right)},$$

where SSIM(X,Y) is the structural similarity index between matrix X and matrix Y representing two images; $\mu_{X}$ is the average mean of the pixels of image X; $\mu_{Y}$ is the average mean of the pixels of image Y; l is the dynamic range of the pixel values; $\sigma_{XY}$ is the covariance of image X and image Y; $\sigma_{X}$ is the variance of image X; and $\sigma_{Y}$ is the variance of image Y.

Moreover, to estimate the quality of the image restoration, we used the peak signal-to-noise ratio (PSNR), described by the following equation [74]:

(16) $$\mathrm{PSNR}=10\log_{10}\left(\frac{{\left(2^{a}-1\right)}^{2}}{\frac{1}{h\times w}\sum_{i=1}^{h}\sum_{j=1}^{w}{\left|{\hat{y}}_{i,j}-y_{i,j}\right|}^{2}}\right),$$

where a is the bit per pixel; h is the height of the image; w is the width of the image; i and j are counters; ${\hat{y}}_{i,j}$ is the pixel value in the position *i* and *j* of the original image; and $y_{i,j}$ is the pixel value in the position *i* and *j* of the denoised image. In our investigation, Equation (16) includes a=8, h=100, w=100. The higher the PSNR, the lower the distortion level.

In our study, we compared the performance of the suggested model of the dual autoencoder with the performance of one autoencoder; these models were built with separable convolutional neural networks and convolutional neural networks. The aim of comparing the separable convolutional neural network with the convolutional neural network is to prove that the separable convolution performs as good as convolutional neural networks in both a dual autoencoder and one autoencoder, and that researchers could rely on them in this field of study where they give the same results or even better results with less computational cost and less trainable parameters, which saves time and memory. When using separable convolutional neural networks and convolutional neural networks, the autoencoder shown in Figure 3 takes a noisy image as an input of size 100 × 100 and contains six layers. The size of filters in the layers except the last layer equals 3 × 3. The first layer contains 50 kernels of filters and one stride, giving an output of the size 98 × 98 × 50; the second layer consists of 25 kernels and two strides, giving an output with a size of 48 × 48 × 25; the third layer consists of 25 kernels and two strides, giving an output of size 23 × 23 × 25. The fourth layer is a transpose layer with 25 kernels and two strides, giving an output of the size 47 × 47 × 25. The fifth layer is a transpose layer with 25 kernels and two strides, giving an output of size 95 × 95 × 25 and the last layer is a transpose layer with one kernel of the size 6x6 and one stride giving the final output as 100 × 100 to match the input size. For the use of separable convolutional neural networks and convolutional neural networks, the dual autoencoder shown in Figure 4 takes a noisy image as an input of size 100 × 100 and consists of twelve layers, six layers for the first encoder and six for the second encoder. The first six layers are described above. The seventh layer consists of 25 kernels with a size of 3 × 3 with one stride giving an output of 98 × 98 × 25. The eighth layer consists of 25 kernels with a size of 3 × 3 and one stride, giving an output with a size of 96 × 96 × 25; the ninth layer consists of 50 kernels of the size of 3 × 3 and two strides, giving an output of size 47 × 47 × 50. The tenth layer is a transpose layer with 50 kernels with a size of 3 × 3 and two strides, giving an output of the size 95 × 95 × 50. The eleventh layer is a transpose layer with 25 kernels with the size of 3 × 3 and one stride, giving an output of size 97 × 97× 25. The last layer is a transpose layer with one kernel of the size 4x4 and one stride, giving the final output as 100 × 100.

The learning process was supervised learning, where the input for each model was the training noised dataset, and the targeted output was the original images’ training dataset. For each model, the training process consisted of 40 epochs with a batch size of 200, and for the backpropagation in the training process, we used a binary cross entropy loss function, and an Adam optimizer with 0.001 as a learning rate. The evaluation was on the testing dataset, and the denoised output of the model after training was compared with the original images’ testing dataset using the structural similarity index (SSIM) and the peak signal-to-noise ratio (PSNR).

**Figure 5:**
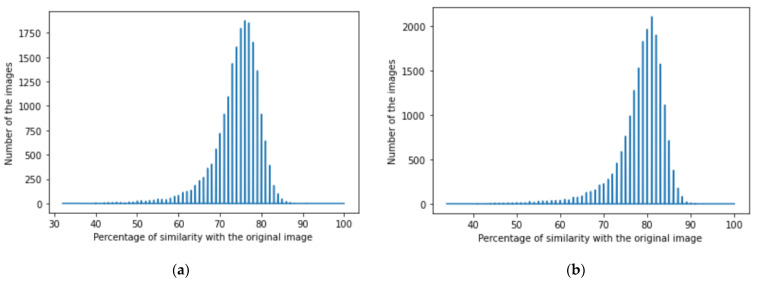
The histograms of similarity between the predicted denoised image and the original image in the case of Gaussian noise: (**a**) one autoencoder with convolutional neural networks; (**b**) dual autoencoder with convolutional neural networks.

**Figure 6:**
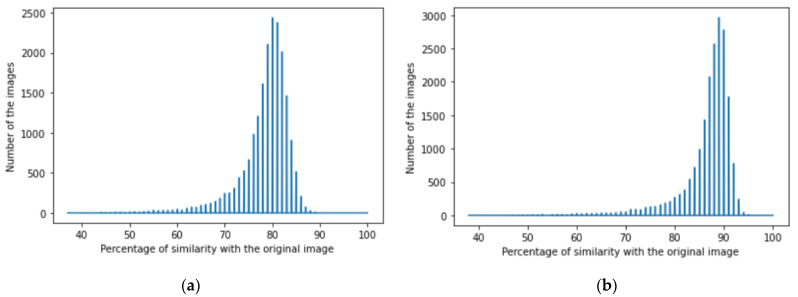
The histogram of similarity between the predicted denoised image and the original image in the case of Gaussian noise: (**a**) one autoencoder with separable convolutional neural networks; (**b**) dual autoencoder with separable convolutional neural networks.

**Figure 7:**
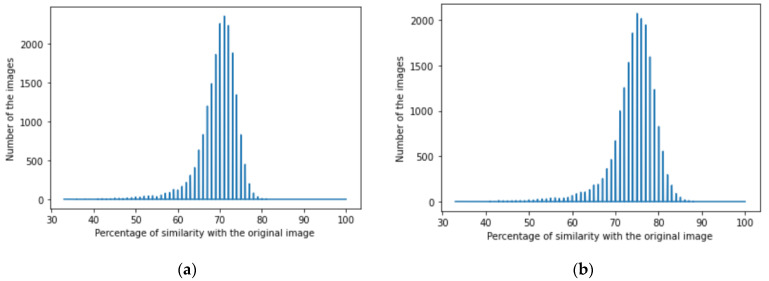
The histogram of similarity between the predicted denoised image and the original image in the case of Poisson noise: (**a**) one autoencoder with convolutional neural networks; (**b**) dual autoencoder with convolutional neural networks.

**Figure 8:**
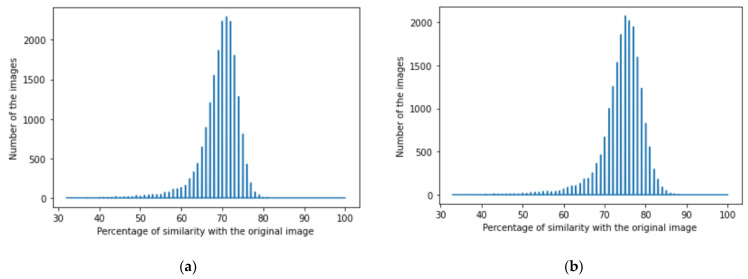
The histogram of similarity between the predicted denoised image and the original image in the case of Poisson noise: (**a**) one autoencoder with separable convolutional neural networks; (**b**) dual autoencoder with separable convolutional neural networks.

**Figure 9:**
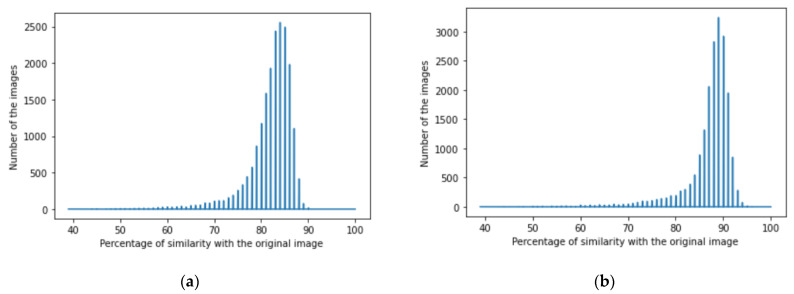
The histogram of similarity between the predicted denoised image and the original image in the case of speckle noise: (**a**) one autoencoder with convolutional neural networks; (**b**) dual autoencoder with convolutional neural networks.

**Figure 10:**
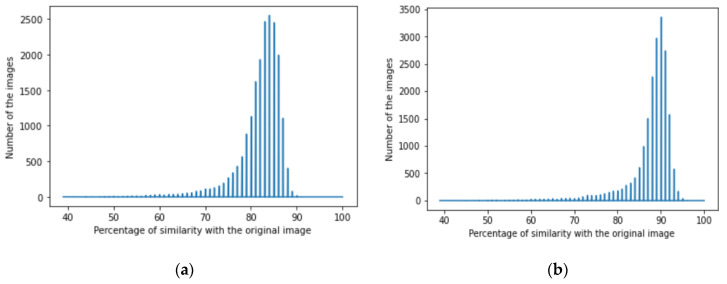
The histogram of similarity between the predicted denoised image and the original image in the case of speckle noise: (**a**) one autoencoder with separable convolutional neural networks; (**b**) dual autoencoder with separable convolutional neural networks.

**Figure 11:**
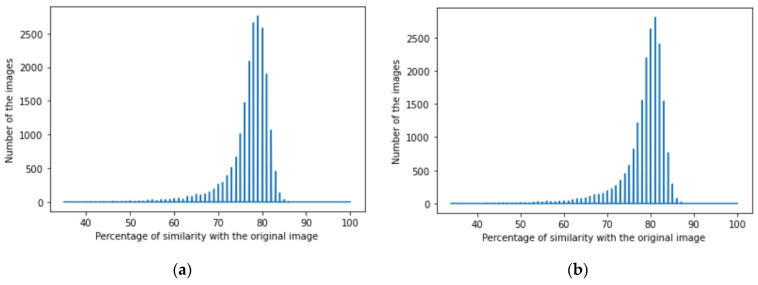
The histogram of similarity between the predicted denoised image and the original image in the case of impulse noise: (**a**) one autoencoder with convolutional neural networks; (**b**) dual autoencoder with convolutional neural networks.

**Figure 12:**
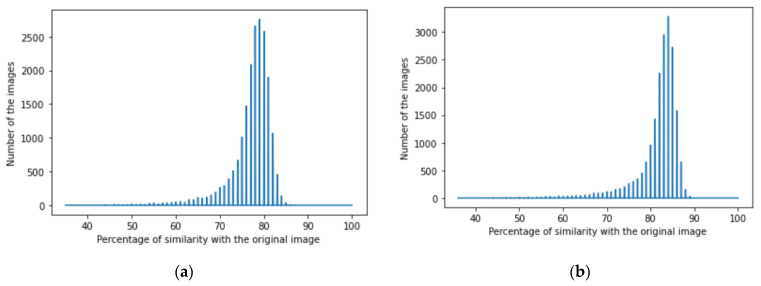
The histogram of similarity between the predicted denoised image and the original image in the case of impulse noise: (**a**) one autoencoder with separable convolutional neural networks; (**b**) dual autoencoder with separable convolutional neural networks.

We achieved image denoising for the testing dataset using the trained model; then, the images predicted by the model were compared with the original images before adding noise using the structural similarity index (15). We presented the results as a histogram of the structural similarity index (15) for the testing data, which shows the accuracy of our noise canceling as a percentage of the similarity knowing that the similarity of noisy images with the original images was zero percent., The histograms of similarity for one autoencoder and a dual autoencoder based on a convolutional neural network (Figure 5a,b) and separable convolutional neural network (Figure 6a,b) when canceling Gaussian noise (Gaussian blur) are depicted below. Corresponding histograms of similarity are shown in Figure 7 and Figure 8, when canceling Poisson noise, in Figure 9 and Figure 10, when canceling speckle noise, and in Figure 11 and Figure 12, when canceling impulse noise.

**Figure 13:**
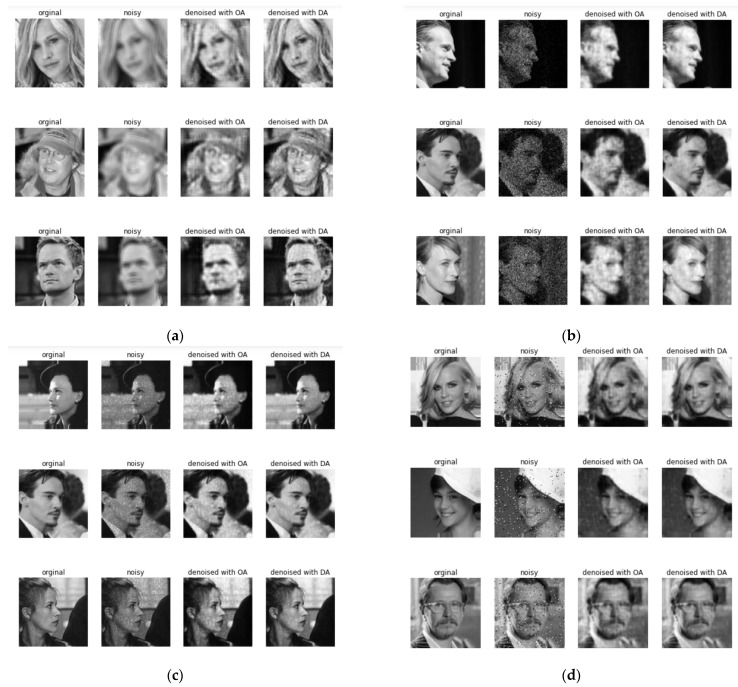
Original image [68,69,70,71], noisy image, and images restored by one and dual autoencoders with separable convolutional neural networks under (**a**) Gaussian noise, (**b**) Poisson noise, (**c**) speckle noise, and (**d**) impulse noise.

The examples of the original image [8,68], the noisy image, and the images restored by one autoencoder (OA) and dual autoencoder (DA) with separable convolutional neural networks are shown in Figure 13a–d under Gaussian noise, Poisson noise, speckle noise, and impulse noise, respectively.

For one autoencoder and a dual autoencoder, the parameter numbers of the convolutions and separable convolutions, the total time of training 80,000 images during 40 epochs, the time of denoising of 19,538 images, the mode *M* of similarities, and the average PSNR after 40 epochs of training for the 19,538 testing images are noted in Table 1, Table 2, Table 3 and Table 4 in the cases of Gaussian noise, Poisson noise, speckle noise, and impulse noise, respectively. In the tables, the statistical mode *M* (%) was the most frequent value of the similarity index percentage between the 19,538 denoised images and the original.

As follows from the analysis of Figure 5, Figure 6, Figure 7, Figure 8, Figure 9, Figure 10, Figure 11 and Figure 12 and Table 1, Table 2, Table 3 and Table 4, the use of a dual autoencoder with separable convolutional neural networks for image denoising gave the following results:

- Image denoising for Gaussian noise (Gaussian blur) with a dual autoencoder achieved better results with both convolutional and separable convolutional neural networks where it raised the mode of the similarities of the testing dataset with the original by approximately 5% for convolution layers and 9% for separable convolution layers and the average PSNR by approximately 8 dB for convolution layers and 9 dB for separable convolution layers. A dual autoencoder with separable convolution layers achieved the higher accuracy with a value of 89% and with a high rate of performance, spending 0.019 s to denoise each image.
- Image denoising for Poisson noise with a dual autoencoder achieved better results for both the use of a convolutional neural network and separable convolutional neural network. Both kinds of neural networks achieved similar accuracy. However, the separable convolution layers enabled it faster with a lower number of parameters.
- Image denoising for speckle noise with a dual autoencoder raised the mode of the accuracies by approximately 6%, where a separable convolutional neural network and convolutional neural network achieved the same results, but the performance of the separable neural network was faster. The average PSNR increased by approximately 2 dB for convolution layers and 4 dB for separable convolution layers. In the dual autoencoder, it took 0.02 s to denoise each image.
- Image denoising for impulse noise due to a dual autoencoder with both a convolutional neural network and separable convolutional neural network raised the *M* by 6% and the average PSNR by approximately 3 dB. Both kinds of neural networks achieved the same accuracy, but the separable convolutional neural network performed faster, where it took 0.023 s to denoise each image.

The research results in the following:

- A dual autoencoder ensures higher accuracy on increasing the total time of training and the time of denoising in comparison with one autoencoder. Thus, to reach a high accuracy of image denoising, we should use a more complicated device and spend a higher computational cost;
- In the mentioned situation, it would be better to have the approach that decreases computational cost without reducing the accuracy of restoring images. This way is a separable convolution in neural networks. In the cases of Poisson noise, speckle noise, and impulse noise, we kept the accuracy gained by the dual autoencoder and got a higher performance of this device by means of separable convolutional neural networks. In the case of Gaussian noise (Gaussian blur), we obtained both a higher accuracy and performance of the device, which is built on the basis of separable convolutional neural networks.

## 5. Discussion

Images frequently symbolize and say something of significance. They hold considerable information and are used for vast applications in security, scientific research, medicine, control, and other fields. In order for images to be beneficial data for these applications, they should be clear and clean from any noise. Therefore, our aim in this study was to solve this fundamental problem, which is image denoise and the restoration of the original images. In image processing and computer vision, we achieved a cancelation of Gaussian noise (Gaussian blur), Poisson noise, speckle noise, and impulse (salt and pepper) noise. To fulfill denoising, we proposed combining two autoencoders together to build a dual autoencoder, where the first autoencoder compresses the input image to approximately 23% of its original size and restores it while training to cancel the different noises, and the second autoencoder works as an enhancer that compresses the image to approximately 50% of its original size and restores it as the final output. Moreover, we used separable convolution layers to build the autoencoders for solving the denoising problem and compared them with the convolutional neural network to prove the ability of the separable convolutional neural network to maintain the same performance as the convolutional neural network and achieve better results while decreasing the number of learnable parameters and the processing time. From comparison with other types of neural networks, we highlight the following advantages of an autoencoder network:

- The ability of feature extraction from non-linear processes and performing non-linear transformations, for instance, image denoising.
- The ability of dimensionality reduction in the autoencoder network process of compressing the data and the feature of information retrievals in the autoencoder network process of decompressing the data.
- Reducing the training parameters decreases the training and performing time of the autoencoder’s network and saves more memory on the user device.

The study’s results show how the use of separable convolution layers in building autoencoders in the field of image processing and image denoising extends the opportunities for better quality performance, presented with the high similarity between the denoised images and the original images, lower complexness, and fast processing. At the same time, a dual autoencoder with separable convolutional neural networks has a relationship design created as a grid of nodes where each pixel in the layer is associated with all the surrounding pixels. This design delivers feature extraction capability, permitting the pixels in the image to be defined as spatial with the locally and equally likely to occur extracted features at any position in the noisy input image. With the proceeds in the advancement of deep learning and the utilization of autoencoders as a model for finding distinctive solutions for specialized issues in image processing and computer vision, in addition to the need to decrease the processing time and memory utilization in numerous gadgets and devices such as microprocessors, phones, or pads, the utilization of separable convolutional neural networks in dual autoencoders will be extraordinary. Indeed, a dual autoencoder increases the accuracy of restoring images; however, it makes the process of encoding and decoding take twice the time of ordinary autoencoders. The separable convolutional neural network decreases the sum of learnable parameters and processing time to adjust the computation cost while achieving high quality and stability in a dual autoencoder.

## 6. Conclusions

In this study we used dual autoencoders for image denoising, which enhance the quality of the denoise where the first autoencoder works as a main image denoiser and the second completes the denoising process and enhances the quality of the denoised images. The use of a separable convolutional neural network in the field of building autoencoders for image processing and image denoising gives a higher performance of devices in comparison with a convolutional neural network. Moreover, for images affected by Poisson noise, speckle noise, and impulse noise, separable convolutional neural networks give the same accuracy of denoising as ordinary convolutional neural networks and better results in the case of Gaussian noise. With the continuous development of deep learning and the use of autoencoders as a tool for finding different solutions for technical problems in image processing and computer vision, and with the need to reduce processing time and memory usage in different devices such as processors, telephones, or pads, the use of a separable convolutional neural network will be signifiable, especially with dual autoencoders, since a dual autoencoder duplicates the process of encoding and decoding. Moreover, separable convolutional neural networks decrease the amount of learnable parameters and processing time to balance the computation cost while achieving high quality results.

## Figures and Tables

**Table 1:** Gaussian noise reduction.

Device of Image Denoising	Neural Network	Number of Parameters	Total Time of Training (s)	Time of Denoising(s)	*M* (%)	Average PSNR (dB)
Autoencoder	CNN	29,626	24,085	446	76	24.52
	SCNN	14,910	20,804	167	80	25.53
Dual Autoencoder	CNN	81,052	54,757	440	81	32.79
	SCNN	51,595	43,038	374	89	34.60

**Table 2:** Poisson noise reduction.

Device of Image Denoising	Neural Network	Number of Parameters	Total Time of Training (s)	Time of Denoising(s)	*M* (%)	Average PSNR (dB)
Autoencoder	CNN	29,626	22,001	60	71	27.38
	SCNN	14,910	19,412	58	71	27.77
Dual Autoencoder	CNN	81,052	56,324	55	76	30.59
	SCNN	51,595	41,207	49	77	31.11

**Table 3:** Speckle noise reduction.

Device of Image Denoising	Neural Network	Number of Parameters	Total Time of Training (s)	Time of Denoising(s)	*M* (%)	Average PSNR (dB)
Autoencoder	CNN	29,626	22,348	209	84	54.48
	SCNN	14,910	19,360	161	84	54.62
Dual Autoencoder	CNN	81,052	53,964	457	89	56.02
	SCNN	51,595	40,912	398	90	58.93

**Table 4:** Impulse noise reduction.

Device of Image Denoising	Neural Network	Number of Parameters	Total Time of Training (s)	Time of Denoising(s)	*M* (%)	Average PSNR (dB)
Autoencoder	CNN	29,626	24,052	184	78	54.93
	SCNN	14,910	20,293	173	79	55.07
Dual Autoencoder	CNN	81,052	55,708	480	85	58.34
	SCNN	51,595	42,007	460	85	58.03

## Data Availability

Data available in publicly accessible repositories those do not issue DOIs. This data can be found here: IMDB-WIKI dataset is available at https://data.vision.ee.ethz.ch/cvl/rrothe/imdb-wiki/ (accessed on 8 July 2022), FER-2013 dataset is available at https://www.kaggle.com/msambare/fer2013 (accessed on 8 July 2022), KDEF dataset is available at https://www.kdef.se/download-2/index.html (accessed on 8 July 2022). Data available on request due to restrictions on use only for non-commercial research and educational purposes. This data can be found here: AffectNet dataset is available at http://mohammadmahoor.com/affectnet/ (accessed on 8 July 2022).
